# Histopathological Patterns and Outcomes of Triple-Positive Versus Triple-Negative Breast Cancer: A Retrospective Study at a Tertiary Cancer Center

**DOI:** 10.7759/cureus.42389

**Published:** 2023-07-24

**Authors:** Mai S Kadi, Alhasan H Alhebshi, Alaa A Shabkah, Walaa A Alzahrani, Ghada N Enani, Ali A Samkari, Omar Iskanderani, Abdulaziz M Saleem, Ali H Farsi, Nora H Trabulsi

**Affiliations:** 1 Department of Community Medicine, Faculty of Medicine, King Abdulaziz University, Jeddah, SAU; 2 Department of Surgery, International Medical Center, Jeddah, SAU; 3 Department of Pediatrics, King Faisal Specialist Hospital and Research Center, Jeddah, SAU; 4 Department of Surgery, Faculty of Medicine, King Abdulaziz University, Jeddah, SAU; 5 Department of Radiology, Faculty of Medicine, King Abdulaziz University, Jeddah, SAU

**Keywords:** progression-free survival, overall survival (os), triple-negative breast carcinoma, breast cancer outcomes, breast cancer research

## Abstract

Background

One of the leading causes of cancer-related deaths in females under 45 years old is breast cancer (BC). The definition of triple-negative breast cancer (TNBC) is the lack of expression of estrogen receptors (ERs) as well as progesterone receptors (PRs) and Erb-B2 receptor tyrosine kinase 2 (HER2) gene amplification. Triple-positive breast cancer (TPBC), on the other hand, is defined as tumors expressing a high level of ER, PR, and HER2 receptors. This study aims to assess the phenotypes of TNBC and TPBC by comparing their individual clinical behavior patterns and prognosis throughout the course of the disease in a tertiary cancer center in the Kingdom of Saudi Arabia (KSA).

Methods

Our study is a retrospective study using electronic medical records (EMRs) to identify all female patients diagnosed with BC using the International Classification of Diseases-10 (ICD-10) codes (between C50 and C50.9). About 1209 cases with primary BC female patients were recognized based on histopathology reports. Further subclassification into TPBC and TNBC was performed. Statistical analysis was performed using Rv3.6.2 (R Studio, version 3.5.2, Boston, MA, USA). The descriptive data were presented as means and standard deviations (SD). Survival curves were approximated using the Kaplan-Meier method. The comparison between survival curves between both groups was achieved using the log-rank test. The multivariate model was constructed based on the identified predictors using univariate analysis.

Results

Univariate analysis of overall survival (OS) showed that mortality was higher in TNBC compared to TPBC (HR = 2.82, P-value <0.05). However, in a multivariate analysis, molecular subtypes did not show a significant effect on OS with a P-value of 0.94. We found that age at diagnosis has been associated with a 4% increase in mortality risk with a yearly rise in age.

Conclusion

In this limited retrospective cohort study, we found that TNBC may not be associated with a higher risk of death than TPBC. However, other factors, including age at diagnosis, surgical intervention, and lymphovascular invasion (LVI), have been observed to increase the risk of mortality. On the other hand, patients with TNBC were found to have a worse prognosis in terms of local recurrence. This information cannot be generalized to all patients with BC given the limitations of this study. Further, larger cohorts are needed to explore biological and treatment-related outcomes in patients with TNBC and TPBC.

## Introduction

One of the leading causes of cancer-related deaths in females under 45 years old is breast cancer (BC) [1]. Recently, the incidence of BC has increased by 0.5% per year worldwide and is expected to rise significantly in the coming years [1].

Multiple factors have been linked to BC etiology, including genetics, environmental, and hormonal factors [2]. Molecular analysis has led to a better understanding of changes at the level of the molecular phase with tumor progression and development [3]. In the modern era, multiple genes are found to express specific hormonal receptors and are linked to BC clinical outcomes [4]. These hormonal receptors' status and genetic predisposition can help further classify BC and guide the treatment plan [3].

The definition of triple-negative breast cancer (TNBC) is the lack of expression of estrogen receptors (ERs) as well as progesterone receptors (PRs) and Erb-B2 receptor tyrosine kinase 2 gene amplification (HER2) [5]. It accounts for 10-15% of all BC and is more common in female patients 40 years old or less and who are of black ethnicity or BRCA1 mutation-positive [6]. It is one of the aggressive types of tumor subtypes, with a rapid growth rate and high likelihood of metastases at the period of diagnosis, along with a high recurrence rate [6].

On the other hand, triple-positive breast cancer (TPBC) is defined as a tumor expressing high levels of ER, PR, and HER2 receptors [7]. This subtype shows different behaviors and responses to therapy and represents 10% of all BC [8]. These tumors react favorably to hormonal treatment and specific targeted therapy [7]. Thus, a better prognosis and significant improvement in survival [7-10].

This study aims to assess the phenotypes of TNBC and TPBC by comparing their individual clinical behavior patterns and prognosis throughout the course of the disease in a tertiary cancer center in the Kingdom of Saudi Arabia (KSA).

## Materials and methods

Study design

Our study is a retrospective study using electronic medical records (EMRs) to identify all female patients diagnosed with BC using the ICD-10 codes, which stand for International Classification of Diseases, tenth version (all codes between C50 and C50.9), at the King Abdulaziz University Hospital in the Kingdom of Saudi Arabia. The Institutional Review Board of KAUH, a tertiary oncology center in the Kingdom of Saudi Arabia (KSA), issued ethical approval for the study (Reference No. 549-22). 

Study population and data collection

We included all female patients diagnosed with BC at our institution with TNBC or TPBC from January 1, 2010 to June 30, 2017. We excluded all other patients with different molecular subtypes. Demographic and cancer-related data, including age at diagnosis, site, clinical presentation, histopathological characteristics, local or distant recurrence, and surgical interventions, were extracted.

Molecular classification

One thousand two hundred nine cases with primary BC female patients were identified based on histopathology reports. Furthermore, we subclassified the patients according to their molecular classification. ER or PR is considered positive if there are at least 1% immunoreactive cancer cell nuclei present, and a sample is considered negative if <1% of cancer cell nuclei are classified as immunoreactive.

The expression status of the HER2 receptor follows a scoring system. A score of 0 and 1+ is considered negative, a 2+ score is equivocal, and it is considered positive if the score is 3+. In the current study, the borderline score 2+ was regarded as negative unless it was verified positive by fluorescent in situ hybridization analysis also known as FISH [11].

Furthermore, tumor grade can be used as an indicator for proliferative function, as determining Ki67 status was not considered standard in our facility in previous years. Subtypes are defined as luminal A, which is ER/PR+, HER2−, and G1/2; luminal B is identified as ER/PR+, HER2−, and G3; TPBC is identified as ER/PR+, HER2+; HER2-enriched status is ER/PR− and HER2+; and TNBC is classified when all receptors are negative ER/PR− and HER2− [12].

Study objective

Our research aims to describe the clinical behaviors, overall survival rate (OS), and progression-free survival rate (PFS) of two distinct subtypes of BC: TNBC and TPBC.

Statistical analysis

The statistical analysis was performed using R Studio, version 3.5.2 (R Studio, version 3.5.2, Boston, MA, USA), which originated in Boston, United States of America, also known as Rv3.6.2. The descriptive data were presented as means and standard deviations (SD). Survival curves were approximated using the Kaplan-Meier method. The comparison between survival curves between both groups was achieved using the log-rank test. Assessing the factors associated with OS and PFS was performed using the univariate Cox proportional hazard (CPH) model. Significant predictors (P<0.1) factor result has been used in the multivariate CPH model to create a model that can foresee survival post-diagnosis. The variables were eliminated stepwise based on clinical judgment and the level of significance to create the final model. CPH does not assume an underlying distribution for the survival function, which offers some advantages compared to parametric survival analyses that require the specification of an underlying survival function. Calculation of both the adjusted hazard ratio (HR) and the associated 95% confidence interval was performed for each predictor in the final model.

HR greater than 1 indicated that the predictors' risk of OS or PFS was considered higher compared to the reference group, while HR <1 indicated lower risk. A 5% level of significance rate was achieved for hypothesis testing.

## Results

Descriptive analysis

Out of the total of 1209 BC female patients diagnosed at KAUH over a 6.5-year interval from 2010 until 2017, we included 276 patients that met the inclusion criteria. Among those, 152 were considered TNBC, and 124 were considered TPBC. Regarding the age at diagnosis, both subtypes had very similar ages at diagnosis, with the mean age at diagnosis in TNBC being 48.7 years (SD: 12.1) and TPBC being 50.9 years (SD: 12.7). Body mass index (BMI) was elevated in the two categories, with a mean BMI of 30.2 (SD: 6.49) and 29.7 (SD: 6.55) for TNBC and TPBC, respectively. The baseline histopathological characteristics of the TNBC and TPBC groups are shown in Table 1.

**Table 1:** The histopathological baseline characteristic of TNBC and TPBC. TPBC: triple-positive breast cancer, TNBC: triple-negative breast cancer, SLNB: sentinel lymph node biopsy, ALND: axillary lymph node dissection, SD: standard deviation.

Variables	TPBC	TNBC	P-value
Tumor size in cm (SD)	3.26 (2.41)	3.82 (2.34)	0.079
Pathological subtype			
Invasive ductal carcinoma	100 (80.6%)	149 (98.0%)	<0.001
Ductal carcinoma in situ	86 (69.4%)	53 (34.9%)	<0.001
Invasive lobular carcinoma	9 (7.26%)	3 (1.99%)	0.067
Lobar carcinoma in situ	9 (7.26%)	0 (0.00%)	0.001
Medullary carcinoma	2 (1.61%)	11 (7.38%)	0.052
Mucinous carcinoma	2 (1.61%)	0 (0.00%)	0.204
Subtypes of ductal carcinoma in situ			
Comedo	47 (37.9%)	27 (17.8%)	<0.001
Cribriform	37 (29.8%)	10 (6.58%)	<0.001
Papillary	3 (2.42%)	2 (1.32%)	0.660
Micropapillary	11 (8.87%)	2 (1.32%)	0.008
Solid	50 (40.3%)	29 (19.1%)	<0.001
Histological grade			0.089
Grade-1: well-differentiated tumor	9 (7.56%)	3 (2.08%)	
Grade-2: moderately differentiated tumor	39 (32.8%)	45 (31.2%)	
Grade-3: poorly differentiated tumor	71 (59.7%)	96 (66.7%)	
Nuclear pleomorphism			<0.00
Score-1	7 (6.09%)	4 (2.88%	
Score-2	68 (59.1%)	51 (36.7%)	
Score-3	40 (34.8%)	84 (60.4%)	
Mitotic count			<0.001
Score-1	64 (60.4%)	26 (18.7%)	
Score-2	27 (25.5%)	44 (31.7%)	
Score-3	15 (14.2%)	68 (48.9%)	
Margin			0.9
Negative	105 (84.7%)	134 (88.7%)	
Positive	19 (15.3%)	17 (11.3%)	
Neuronal invasion			0.045
No	113 (91.1%)	148 (97.4%)	
Yes	11 (8.87%)	4 (2.63%)	
Lymphatic invasion			0.001
No	67 (54.0%)	99 (65.6%)	
Yes	40 (32.3%)	49 (32.5%)	
Unknown	17 (13.7%)	3 (1.99%)	
Number of lymph nodes involved (SD)	2.83 (5.05)	2.09 (4.41)	0.227
Therapeutic intervention			
Surgical intervention	109 (87.9%)	129 (84.9%)	0.581
Modified radical mastectomy (mastectomy + ALND)	68 (54.8%)	68 (44.7%)	0.121
Lumpectomy + axillary dissection	29 (23.4%)	36 (23.7%)	1.000
Lumpectomy + SLNB	11 (8.87%)	25 (16.4%)	0.093
Distant metastasis and locoregional recurrence			
Distant metastasis	34 (27.4%)	52 (34.2%)	0.280
Site of distant metastasis			
Lung	18 (14.5%)	46 (30.3%)	0.003
Bone	23 (18.5%)	23 (15.1%)	
Liver	13 (10.5%)	24 (15.8%)	0.267
Brain	9 (7.26%)	10 (6.58%)	1.000
Involvement of the contralateral breast	5 (4.03%)	6 (3.95%)	1.000
Recurrence	12 (9.68%)	31 (20.4%)	0.023
Mortality	13 (10.5%)	33 (21.7%)	0.020

The majority of patients in both subtypes had a poorly differentiated histological grade at the time of diagnosis (66.7% and 59.7%) for TNBC and TPBC, respectively. However, TNBC showed more aggressive characteristics, with a nuclear pleomorphism score of 3 in 60.4% of cases compared to 34.8% in TPBC (p<0.001) and a high mitotic count, a score of 3 in 48.9% of patients compared to 14.2% (p<0.001). 

We found that the most common pathological subtype was invasive ductal carcinoma for TNBC and TPBC (98% and 80.6%), respectively (p<0.001). In situ components of ductal carcinoma co-existing with invasive BC were observed less frequently in TNBC 34.9% compared to only 69.4% for TPBC (p<0.001). The most common pattern of ductal carcinoma in situ for both was the solid pattern (40.3% and 19.1%) for TPBC and TNBC, respectively (p<0.001).

Furthermore, lobular carcinoma in situ didn't exist in TNBC compared to 7.26% of TPBC (p-value = 0.001). The tumor size was larger in TNBC compared to TPBC. The mean tumor sizes of TNBC and TPBC were 4.01 cm and 3.26 cm (p-value = 0.043), respectively. The lymphatic invasion was observed more in TNBC (65.6%) than in TPBC (54%) (p-value = 0.001).

Treatment-related characteristics

A total of 84.9% and 87.9% were candidates for surgical intervention in TNBC and TPBC, respectively. Mastectomy was more commonly performed regardless of the molecular subtype rather than breast conservative therapy. Moreover, postoperative local complications, including hematoma, seroma, and wound infection, were observed more frequently in TPBC than in TNBC, with percentages of 15.3% and 6.6%, respectively (p-value = 0.031). TPBC patients received radiotherapy more frequently than TNBC patients, with percentages of 60.8% and 43%, respectively (p-value = 0.005).

Survival outcomes

Regarding the location of metastasis, the lungs were the most common site in the TNBC group 30.3% compared to 14.5% in TPBC with a significant p-value of 0.003. There was a higher rate of recurrence in TNBC 20.4% compared to TPBC 9.68% (p-value = 0.023). Mortality incidence was markedly higher in the TNBC group compared to TPBC 21.7% vs 10.5%, respectively (p-value = 0.002).

Overall Survival

Univariate analysis: The mortality hazard was higher in TNBC compared to TPBC (HR = 2.82, p<0.05). Regarding tumor size, there is a 9% increase in mortality risk with each centimeter increase in tumor size (HR = 1.09, p-value = 0.008). BC associated with lymphovascular invasion (LVI) were at higher risk of mortality compared to those with no lymphovascular invasion (HR = 2.2, p-value = 0.02).

Patients who didn't receive adjuvant chemotherapy were almost at twice higher risk of mortality compared to those who received it (HR = 1.99, p-value = 0.038). The utilization of immunotherapy was associated with a lower hazard of death (HR = 0.21, p<0.05). Patients who didn't receive radiotherapy had a higher risk of death compared to patients who received radiotherapy (HR = 2.34, p-value = 0.009).

**Figure 1:**
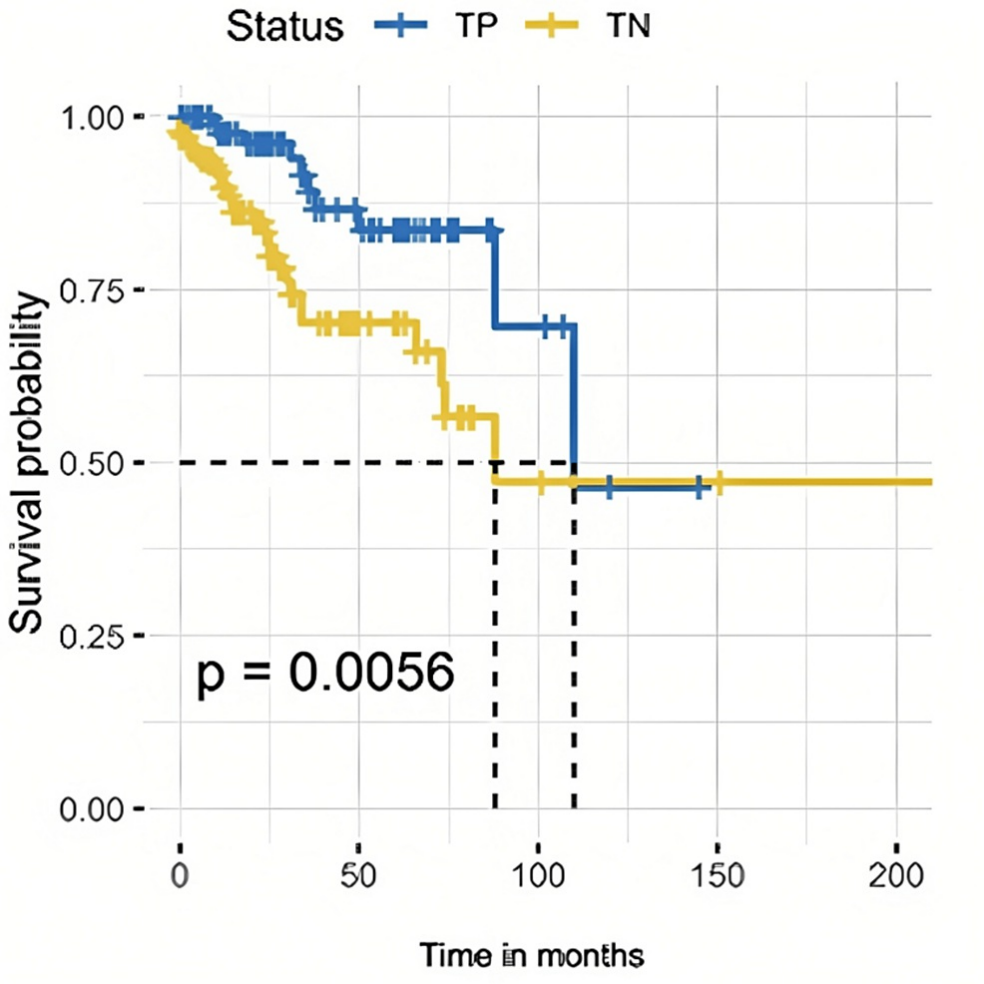
Kaplan-Meier estimates for overall survival. TP: triple-positive breast cancer; TN: triple-negative breast cancer.

The surgical intervention reduced the risk of death by 86% compared to those with no surgical intervention (HR = 0.14, p<0.001). The risk of death was lower with lumpectomy (HR = 0.34, p<0.05). However, mastectomy did not show a significant improvement in survival in univariate analysis (HR = 0.86, p-value = 0.635). Axillary lymph node dissection (ALND) showed a lower risk of death (HR = 0.49, p-value = 0.028). The log-rank test was statistically significant for OS (p-value = 0.0056). Figure 1 shows Kaplan-Meier estimates for OS in TNBC and TPBC.

Multivariate analysis: Regarding age at diagnosis, it was observed that there is a 4% increase in the risk of death with each year's growth in age (HR = 1.04, p-value = 0.02). Positive lymphovascular invasion was associated with an elevated risk of mortality compared to negative lymphovascular invasion (HR = 2.52, p = 0.02). Lumpectomy reduced mortality by 94% (HR = 0.06, p<0.001). Mastectomy was associated with a significant reduction in mortality by 99% (HR = 0.01, p<0.001). There was no association between molecular subtype and OS after adjustment of HR in multivariate analysis (HR = 0.96, p-value = 0.94). Table 2 presents OS along with multivariate CPH regression analysis for each group in terms of HR and p-values.

**Table 2:** Univariate and multivariate Cox proportional hazards regression analysis to estimate overall survival. *P value could not be computed as no events occurred in the + SLN group. TPBC: triple-positive breast cancer, TNBC: triple-negative breast cancer, SD: standard deviation, SLNB: sentinel lymph node biopsy, ALND: axillary lymph node dissection, ILC: invasive lobar carcinoma, IDC: invasive ductal carcinoma, LCIS: lobular carcinoma in situ.

	Univariant analysis of overall survival				Multivariant analysis of overall survival	
	No event	Event	HR	P-value	Hazard ratio	P-value
	N = 228	N = 44				
Age at diagnosis	49.4 (12.1)	51.9 (14.8)	1.02 (1.00;1.05)	0.082	1.04 (1.01;1.07)	0.02
BMI mean (±SD)	30.1 (6.61)	30.2 (7.02)	0.99 (0.94;1.04)	0.663		
Molecular subtype						
TPBC	93 (91.2%)	9 (8.82%)	Ref	Ref	Ref	
TNBC	114 (79.2%)	30 (20.8%)	2.82 (1.34;5.95)	0.006	0.96 (0.35;2.64)	0.94
Neo-adjuvant chemotherapy						
Yes	61 (81.3%)	14 (18.7%)	Ref	Ref		
No	140 (85.9%)	23 (14.1%)	0.69 (0.35;1.36)	0.286		
Radiotherapy						
Yes	136 (86.6%)	21 (13.4%)	Ref	Ref		
No	64 (78.0%)	18 (22.0%)	2.34 (1.24;4.44)	0.009		
Hormonal treatment						
No	120 (82.2%)	26 (17.8%)	Ref	Ref		
Yes	80 (87.9%)	11 (12.1%)	0.51 (0.25;1.03)	0.061		
Immunotherapy						
No	127 (79.4%)	33 (20.6%)	Ref	Ref		
Yes	70 (94.6%)	4 (5.41%)	0.21 (0.07;0.60)	0.003		
Tumor size (cm), mean (±SD)	3.34 (2.24)	5.80 (4.84)	1.09 (1.02;1.16)	0.008		
Number of lymph nodes involved	2.26 (4.59)	3.00 (4.58)	1.02 (0.96;1.09)	0.527		
Adjuvant chemotherapy						
Yes	130 (87.2%)	19 (12.8%)	Ref	Ref		
No	69 (79.3%)	18 (20.7%)	1.99 (1.04;3.82)	0.038		
Lymphovascular invasion						
Negative	135 (88.8%)	17 (11.2%)	Ref	Ref	Ref	Ref
Positive	60 (76.9%)	18 (23.1%)	2.20 (1.13;4.27)	0.020	2.52 (1.19;5.34)	0.02
Unknown	11 (73.3%)	4 (26.7%)	1.68 (0.56;5.06)	0.353	1.17 (0.36;3.87)	0.79
Perineural invasion						
No	198 (84.6%)	36 (15.4%)	Ref	Ref		
Yes	9 (75.0%)	3 (25.0%)	1.46 (0.44;4.81)	0.536		
Histological grade						
Grade-3: poorly differentiated tumor	124 (82.1%)	27 (17.9%)	Ref	Ref		
Grade-2: moderately differentiated tumor	65 (86.7%)	10 (13.3%)	0.75 (0.36;1.55)	0.435		
Grade-1: well-differentiated tumor	7 (87.5%)	1 (12.5%)	0.52 (0.07;3.85)	0.524		
Surgical intervention						
No	20 (60.6%)	13 (39.4%)	Ref	Ref		
Yes	187 (87.8%)	26 (12.2%)	0.14 (0.07;0.28)	<0.001		
Lumpectomy						
No	121 (79.6%)	31 (20.4%)	Ref	Ref	Ref	Ref
Yes	86 (91.5%)	8 (8.51%)	0.34 (0.16;0.74)	0.007	0.06 (0.02;0.16)	<0.001
Mastectomy						
No	106 (83.5%)	21 (16.5%)	Ref	Ref	Ref	Ref
Yes	101 (84.9%)	18 (15.1%)	0.86 (0.46;1.61)	0.635	0.01 (0.0.4;0.25)	<0.001
SLNB						
No	162 (80.6%)	39 (19.4%)	Ref	Ref		
Yes	45 (100%)	0 (0.00%)	0.00 (0.00)	0.996		*
ALND						
No	69 (80.2%)	17 (19.8%)	Ref	Ref		
Yes	138 (86.2%)	22 (13.8%)	0.49 (0.26;0.93)	0.028		

Progression-Free Survival

**Figure 2:**
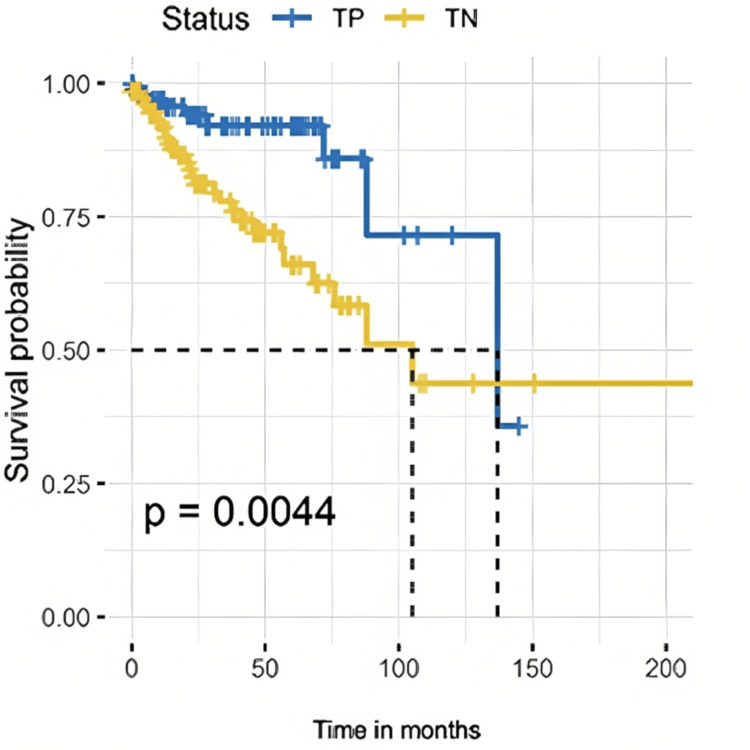
Kaplan-Maier estimates for progression-free survival. TP: triple-positive breast cancer, TN: triple-negative breast cancer.

Univariate analysis: TNBC showed more risk of recurrence in comparison to TPBC (HR = 2.67, p-value = 0.008). A higher number of positive lymph nodes was associated with a 6% increase in the risk of recurrence (HR = 1.06, p-value = 0.038). The risk of recurrence was higher with lymphatic invasion (HR = 2.26, p<0.013). The log-rank test for PFS was statistically significant (p-value = 0.0044). Figure 2 shows Kaplan-Meier estimates for PFS in TNBC and TPBC.

Multivariate analysis: The TNBC molecular subtype was associated with more risk of recurrence in comparison to the TPBC (HR = 2.71, p-value = 0.007). Lymphatic invasion showed an association with more risk of recurrence (HR = 2.41, p-value = 0.008). Table 3 presents the PFS univariate and multivariate CPH regression analyses.

**Table 3:** Univariate and multivariate Cox proportional hazard regression analysis to estimate progression-free survival. TPBC: triple-positive breast cancer, TNBC: triple-negative breast cancer, SD: standard deviation, SLNB: sentinel lymph node biopsy, ALND: axillary lymph node dissection, ILC: invasive lobular carcinoma, IDC: invasive ductal carcinoma, LCIS: lobular carcinoma in situ.

	Univariant analysis of progression-free survival				Multivariant analysis of progression-free survival	
	No event	Event	HR	P-value	Hazard ratio	P-value
	N = 207	N = 38				
Nationality						
Saudi	93 (86.9%)	14 (13.1%)	Ref	Ref		
Non-Saudi	114 (82.6%)	24 (17.4%)	1.53 (0.79;2.96)	0.209		
Age at diagnosis	50.3 (12.6)	46.8 (12.5)	0.98 (0.95;1.01)	0.250		
BMI	30.1 (6.74)	30.3 (6.30)	0.99 (0.94;1.04)	0.678		
Molecular subtype						
TPBC	92 (90.2%)	10 (9.80%)	Ref	Ref	Ref	Ref
TNBC	115 (80.4%)	28 (19.6%)	2.67 (1.29;5.50)	0.008	2.71 (1.31;5.61)	0.007
Neo-adjuvant chemotherapy						
Yes	63 (84.0%)	12 (16.0%)	Ref	Ref		
No	137 (84.6%)	25 (15.4%)	0.97 (0.49;1.93)	0.929		
Radiotherapy						
Yes	130 (82.8%)	27 (17.2%)	Ref	Ref		
No	70 (86.4%)	11 (13.6%)	1.21 (0.60;2.46)	0.593		
Hormonal treatment						
No	119 (82.1%)	26 (17.9%)	Ref	Ref		
Yes	80 (87.9%)	11 (12.1%)	0.49 (0.24;1.00)	0.050		
Immunotherapy						
No	131 (82.4%)	28 (17.6%)	Ref	Ref		
Yes	65 (87.8%)	9 (12.2%)	0.50 (0.24;1.07)	0.074		
Tumor size (cm), mean (±SD)	3.71 (3.11)	3.89 (2.68)	1.01 (0.93;1.10)	0.821		
Number of lymph nodes involved	2.03 (4.15)	4.38 (6.21)	1.06 (1.00;1.12)	0.038		
Adjuvant chemotherapy						
Yes	120 (81.1%)	28 (18.9%)	Ref	Ref		
No	78 (89.7%)	9 (10.3%)	0.70 (0.33;1.50)	0.363		
Lymphatic invasion						
Negative	135 (88.2%)	18 (11.8%)	Ref	Ref	Ref	
Positive	57 (75.0%)	19 (25.0%)	2.26 (1.18;4.32)	0.013	2.41 (1.26;4.60)	0.008
Unknown	14 (93.3%)	1 (6.67%)	0.45 (0.06;3.40)	0.439	0.6 (0.08;4.56)	0.618
Neuronal invasion						
No	198 (85.0%)	35 (15.0%)	Ref	Ref		
Yes	9 (75.0%)	3 (25.0%)	1.48 (0.45;4.85)	0.514		
Histological grade						
Grade-3: poorly differentiated tumor	123 (81.5%)	28 (18.5%)	Ref	Ref		
Grade-2: moderately differentiated tumor	67 (90.5%)	7 (9.46%)	0.50 (0.22;1.16)	0.106		
Grade-1: well-differentiated tumor	7 (87.5%)	1 (12.5%)	0.68 (0.09;5.05)	0.709		
Surgical intervention						
No	33 (97.1%)	1 (2.94%)	Ref	Ref		
Yes	174 (82.5%)	37 (17.5%)	2.30 (0.31;17.1)	0.415		
Lumpectomy						
No	129 (84.9%)	23 (15.1%)	Ref	Ref		
Yes	78 (83.9%)	15 (16.1%)	0.90 (0.47;1.73)	0.745		
Mastectomy						
No	111 (87.4%)	16 (12.6%)	Ref	Ref		
Yes	96 (81.4%)	22 (18.6%)	1.26 (0.66;2.41)	0.481		
SLNB						
No	170 (84.6%)	31 (15.4%)	Ref	Ref		
Yes	37 (84.1%)	7 (15.9%)	1.38 (0.60;3.14)	0.448		
ALND						
No	75 (87.2%)	11 (12.8%)	Ref	Ref		
Yes	132 (83.0%)	27 (17.0%)	0.78 (0.39;1.60)	0.502		
ILC						
No	197 (84.2%)	37 (15.8%)	Ref	Ref		
Yes	10 (90.9%)	1 (9.09%)	0.73 (0.10;5.34)	0.756		
IDC						
No	24 (96.0%)	1 (4.00%)	Ref	Ref		
Yes	183 (83.2%)	37 (16.8%)	4.70 (0.64;34.4)	0.127		
LCIS						
No	200 (84.4%)	37 (15.6%)	Ref	Ref		
Yes	7 (87.5%)	1 (12.5%)	0.61 (0.08;4.42)	0.621		

## Discussion

BC remains the most common cancer in women aged 40 years or older in KSA. Over fifty percent of BC cases in KSA are detected at an advanced stage, compared to 20% in developed countries [2]. This results in a higher mortality rate, a lower cure rate and higher costs [2].

The age of diagnosis of BC varies where approximately 25% are diagnosed at less than 50 years and 20% of patients are older than 75 years [13]. To our knowledge, this is the first review comparing TNBC and TPBC in terms of behavior and prognosis. In our data, both subtypes had very similar ages at diagnosis, with a mean age of 50.9 in TPBC (SD: 12.7) and 48.7 (SD: 12.1) in TNBC, which is consistent with the mean age at diagnosis found in the literature (the mean age of TPBC is 51 years and TNBC is 49 years) [14,15].

In our study, the univariate analysis of OS showed that the hazard of mortality was higher in TNBC compared to TPBC (HR = 2.82, p<0.05). However, in a multivariate analysis, molecular subtypes did not show a significant effect on OS, with a P-value of 0.94. We found that age at diagnosis has been associated with a 4% increase in mortality risk with a yearly rise in age (HR = 1.04, p-value = 0.02). It has been noticed that patients with TNBC have worse OS and disease-specific survival in comparison to non-TNBC with 60-70% of five-year survival [16]. Molecular subtypes showed marked differences in the rate of OS (P<0.001). The patient with the luminal A subtype has more prolonged survival than the luminal B and HER2 enriched subtypes, respectively. The shortest survival was observed among patients with TNBC [17].

It has been widely reported that TNBC usually has a more aggressive nature than all BC subtypes, as it is usually associated with a high rate of metastasis with a specific pattern of metastasis and also a high rate of disease recurrence, which ultimately affects and decreases the OS. Relapse rates within the first three years are seen more with TNBC along with death rates in the first five years after diagnosis [18]. In our study, TNBC was found to be associated with a higher risk of recurrence compared to TPBC in multivariate analysis (HR = 2.71, p-value = 0.007).

Recent updates suggested the utilization of neoadjuvant systemic therapy in the early stages of TNBC as well as in the late stages of TNBC [19]. These were influenced by a recent result of the KEYNOTE-522 trial in which it showed a larger number of patients who has an early-stage TNBC had a complete pathological response when treated with immunotherapy and neoadjuvant chemotherapy compared with placebo and neoadjuvant chemotherapy [20]. In TNBC, local and distant recurrence occurs more frequently, and locoregional radiotherapy plays a vital role in the treatment of TNBC. Recent trials have studied the use of a mixture of the immune system and radio-immunotherapy to improve the outcomes in TNBC [21].

In our study, the univariate analysis exhibited in OS showed that patients who did not receive adjuvant chemotherapy were almost at twice a higher risk of mortality compared to those who received it (HR = 1.99, p-value = 0.038). The utilization of immunotherapy was linked with a decreased hazard of death (HR = 0.21, p<0.05). Patients who did not receive radiotherapy were at higher risk compared to those who received it (HR = 2.34, p-value = 0.009).

A prospective study involved 32,502 BC cases in which adjuvant chemotherapy was shown to be associated with better OS (HR = 0.75, 95% CI (0.69-0.83), p = 0.0001) and distant disease-free survival (HR = 0.82, 95% CI (0.75-0.90), p = 0.0001), which is similar to the result of our study [22].

The prognosis of BC that exhibits negative hormonal status, for instant TNBC, is greatly influenced by lymphovascular invasion in comparison to positive hormonal status [23]. It has been observed in our study that TNBC was more likely to have lymphatic invasion than TPBC 65.6% vs. 54%, respectively (p-value 0.001). In a prior study, the presence of lymphovascular invasion was linked with worse PFS (p<0.01) and OS (p<0.01). In multivariate models adjusted for the BC subtype, the lymphovascular invasion was largely associated with a lower rate of PFS (p<0.01) and OS (p<0.01) [24]. These findings were concordant with our results. 

With regards to metastasis, TNBC is more complex and is more likely to metastasize with relatively poor outcomes [25]. In some studies, the location of the metastasis is indicated by the HR+ status, where they are most likely to have bone metastasis, whereas HR- tumors tend to have visceral sites metastasized [26]. In our study, the location of metastases was most commonly seen in the lungs for TNBC (30.3%), with a significant p-value = 0.003. In TPBC bone was the commonest site of metastasis with 23 (18.5%) (p-value = 0.552).

TNBC usually presents with a higher tumor grade, a larger tumor size, and a worse prognosis with aggressive tendencies [8,14]. TNBC had a mean size of 4.01 cm, while the mean tumor size was 3.26 cm (with a p-value of 0.043). There was a 9% increase in the risk of mortality with each centimeter rise in tumor size (HR = 1.09, p-value = 0.008). In the literature, a decrease of 1.5 cm was linked with a decrease in the death rate of 23.0% in the positive nodes group and of 10.8% in the negative nodes group [27].

In a recent study, the median OS between the group undergone surgery and the non-surgical group was found to be 30.3 months and 20.7 months, respectively [28]. Another study published in 2021 stated the superiority of breast preservation therapy in terms of lumpectomy with radiation therapy over mastectomy in general and specific to BC in a relative survival increase of 56% to 70% in patients with negative nodes [29]. The same association was seen in weaker-strength patients with positive nodes, but not in females with advanced nodal stages [29]. In our study, having a surgical intervention was associated with an 86% lower risk of mortality compared to those who did not undergo a surgical intervention (HR = 0.14, p<0.001). Lumpectomy was associated with a lower risk of death (HR = 0.34, p<0.05) compared to mastectomy (HR = 0.86, p-value 0.635). Moreover, axillary lymph node dissection (ALND) was linked with a decreased risk of death (HR = 0.49, p-value = 0.028). Furthermore, our multivariate analysis showed a significant reduction in both lumpectomy and mastectomy. Lumpectomy was associated with a significant decrease in mortality rate by 94% (HR = 0.06, p<0.001), while mastectomy was associated with a significant reduction in mortality by 99% (HR = 0.01, p<0.001).

Strength and limitation

This is the first study in the Middle East to compare TNBC with TPBC. However, one of the major study limitations is that the study is retrospective and that we have not looked at important predictors of disease outcome such as treatment modalities received by the patients, which would have required a larger cohort of patients. This may also have affected our ability to identify other relevant predictors. As with any retrospective study, loss of follow-up is a limitation; nevertheless, we made every attempt to contact patients who had not returned for follow-up in the previous two years in order to identify those who had a recurrence, died, or were treated elsewhere. The OS outcomes in our analysis might not correspond to the literature due to the previously mentioned limitations. However, in terms of recurrence, it corresponded to the literature. Thus, due to these limitations, our study results must be interpreted carefully. 

Recommendation 

Prospective studies on a larger scale comparing TNBC with TPBC, pathological patterns and therapeutic approaches are needed.

## Conclusions

In this limited retrospective cohort study, we found that TNBC may not be associated with a higher risk of death than TPBC. However, other factors, including age at diagnosis, surgical intervention, and LVI, have been observed to increase the risk of mortality. On the other hand, patients with TNBC were found to have a worse prognosis in terms of local recurrence. This information cannot be generalized to all patients with BC given the limitations of this study. Further, larger cohorts are needed to explore biological and treatment-related outcomes in patients with TNBC and TPBC. 
